# Risk factors for upper extremity deep vein thrombosis after esophagectomy for esophageal cancer in retrosternal reconstruction

**DOI:** 10.1007/s10388-025-01122-x

**Published:** 2025-04-15

**Authors:** Yusuke Ogawa, Yu Ohkura, Masaki Ueno, Kentoku Fujisawa, Hayato Shimoyama, Shusuke Haruta, Harushi Udagawa

**Affiliations:** 1 Department of Gastroenterological Surgery, Toranomon Hospital, 2-2-2 Toranomon, Minato-ku, Tokyo, 105-8470 Japan; 2 Okinaka Memorial Institute for Medical Research, Tokyo, Japan

**Keywords:** Esophagectomy, Esophageal cancer, Retrosternal reconstruction, Upper extremity deep vein thrombosi**s**

## Abstract

**Background:**

Upper extremity deep vein thrombosis (UEDVT) is a fatal postoperative complication that can cause pulmonary embolism (PE). There have been few reports on the relationship between esophageal cancer and UEDVT. The aim of this study is to analyze the risk factors for UEDVT in esophageal cancer.

**Methods:**

Seventy-five cases of thoracic esophageal cancer who underwent one-stage curative resection and reconstructive surgery from May 2019 to June 2022 were included. The stomach or ileocolon was selected as the reconstructive graft. All cases requiring chemotherapy were treated with a peripheral central venous catheter (PICC). To evaluate the width of the retrosternal space, the retrosternal ratio and the cross-sectional area of the graft intestine were measured at the level of the left brachiocephalic vein.

**Results:**

UEDVT was observed in 11 patients (14.7%) and occurred only with gastric tube reconstruction (*p* = 0.02). The width of the retrosternal space was significantly different between the UEDVT and non-UEDVT groups (*p* = 0.002). The cross-sectional area of reconstructive organ was larger in the stomach than in the ileocolon (*p* < 0.01). Patients with a history of PICC insertion from the left side had a higher incidence of UEDVT (*p* = 0.025).

**Conclusions:**

In esophagectomy, gastric tube reconstruction, a retrosternal ratio less than 0.16, and history of PICC insertion from the left side are risk factors for UEDVT.

## Introduction

Deep vein thrombosis (DVT) and pulmonary thromboembolism (PE) are referred to as venous thromboembolism (VTE). According to the Analysis of National Clinical Database in Japan, esophagectomy has the highest incidence of VTE among gastrointestinal surgeries with a probability of 5.1% [1, 2]. It is said that 60% of postoperative thrombotic events occur within 21 days after surgery; therefore, VTE is a disease frequently encountered in daily clinical practice [3]. Upper extremity deep vein thrombosis (UEDVT) accounts for 11% of DVT and it is thought to be caused by central venous catheters and malignant tumors [4, 5]. The incidence of UEDVT increases 18-fold in the presence of malignant tumors, and it has been reported that 36% of UEDVT cases are associated with PE [6]. In addition, the in-hospital mortality rate due to PE in patients with carcinoma has been reported at 11.8% [7, 8]. Therefore, UEDVT is a fatal complication that should be avoided from the early postoperative period. However, there is very little literature on UEDVT that occurs after esophagectomies, and there are no reports on the association between UEDVT and ileocolonic reconstruction. Here, we aimed to clarify the risk factors for the development of UEDVT after esophagectomy for esophageal cancer.

## Patients and methods

### Study population

We performed 164 esophagectomies for esophageal cancer from May 2019 to June 2022. These patients underwent blood sampling, upper gastrointestinal endoscopy, contrast-enhanced CT (CECT), PET/CT, abdominal ultrasonography, and cervical ultrasonography as preoperative examinations. For patients who were eligible for neoadjuvant chemotherapy, one to three courses of 5-fluorouracil and cisplatin (FP) regimen or docetaxel, cisplatin, and 5-fluorouracil (DCF) regimen were administered [9]. To avoid complications caused by extravasation of anticancer drugs, a hospital policy was implemented to insert a peripheral central venous catheter (PICC) in all cases.

**Fig. 1:**
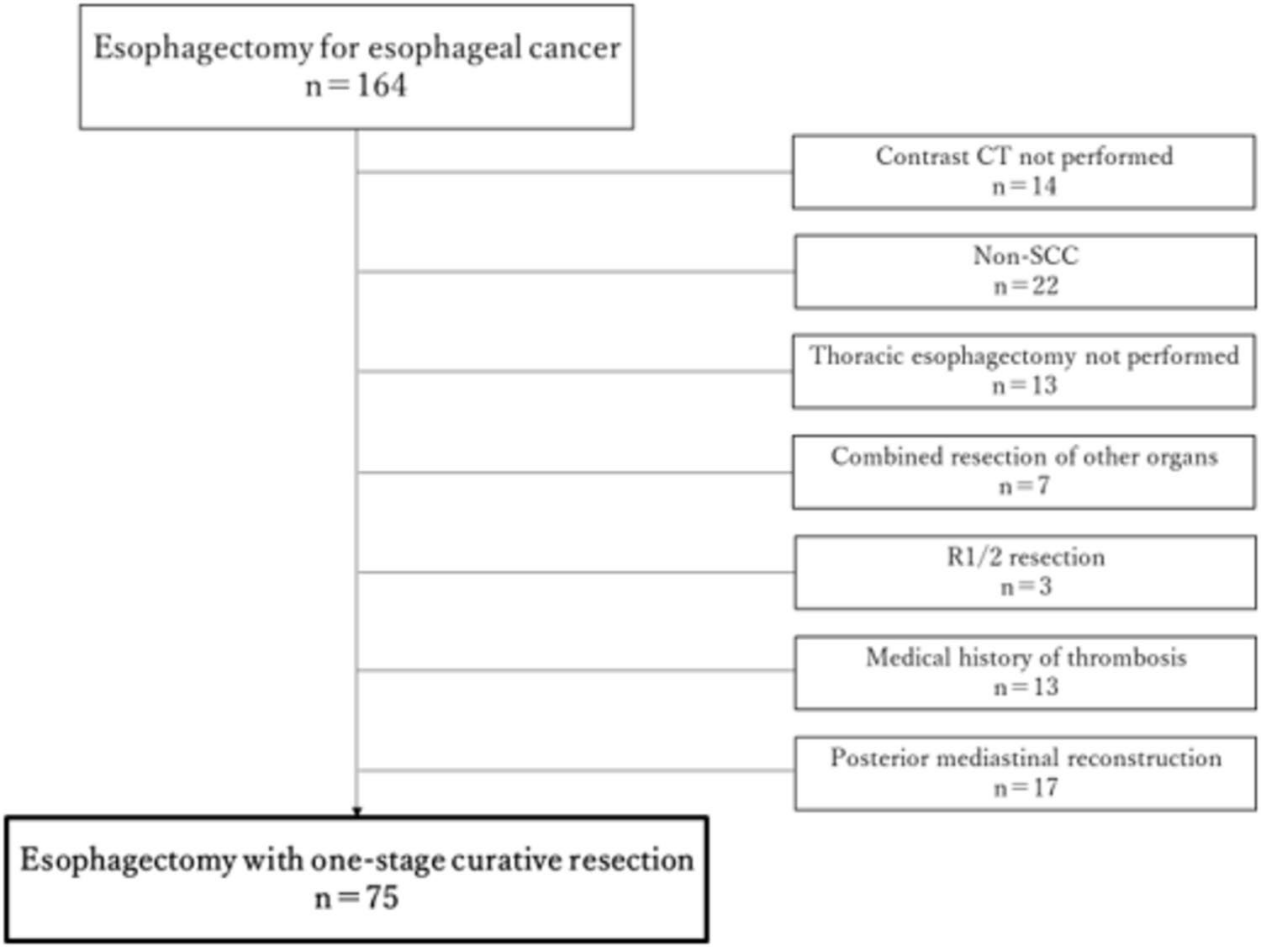
A total of 75 patients who underwent curative esophagectomy for esophageal cancer from May 2019 to June 2022. *SCC* squamous cell carcinoma

Of these 164 patients, cases in which CECT could not be performed due to renal dysfunction, drug allergy or other reasons, cases in which the histological tumor type was not squamous cell carcinoma, cases in which thoracic esophagectomy was not performed, because the main lesion was located in the cervical esophagus or esophagogastric junction, cases in which combined resection of other organs were performed, cases in which R0 resection was not performed, cases in which antithrombotic drugs were administered perioperatively due to a medical history of thrombosis, and cases in which posterior mediastinal reconstruction was performed were excluded (Fig. 1). Therefore, 75 patients with thoracic esophageal squamous cell cancer who underwent one-stage curative resection and reconstructive surgery were included in this study (Table 1).

**Table 1:** Clinicopathological characteristics of patients who underwent curative esophagectomy

Variable	UEDVT group (n = 11)	non-UEDVT group (n = 64)	Univariate *P* value
Age	64 ± 7.6	66 ± 8.8	0.669
Gender (Male/Female)	8/3	49/15	0.523
BMI	20.1 ± 3.4	21.8 ± 3.2	0.055
Tumor location			
Ut/Mt/Lt	3/2/6	7/28/29	0.167
cStage			
I/II/III/IV	3/2/2/3	27/15/19/3	0.084
Neoadjuvant chemotherapy			
Yes/No	7/4	31/33	0.352
Thoracic operative approach			
Robot-assisted	8	35	0.456
Video-assisted thoracoscopic	3	25	
Open	0	4	
Abdominal operative approach			
Laparoscopic	5	32	0.961
Hand-assisted laparoscopic	4	21	
Open	2	11	
Lymph-node dissection			
3-field/2-field	9/2	35/29	0.085
Intestinal graft			
Stomach/ileocolon	11/0	43/21	**0.02**
Cross-sectional area of Intestinal graft			
Stomach	856.51 ± 270.5	840.34 ± 259.2	0.855
Ileocolon	–	351.0 ± 221.0	–
Retrosternal ratio	0.148 ± 0.05	0.233 ± 0.06	**0.002**
Server complications (Clavien–Dindo Grade≧IIIb)			
Yes/No	1/10	4/60	0.558

*P* values were calculated by Mann–Whitney *U* test or Pearson’s chi-square test

*BMI* body mass index, *Ut* upper thoracic esophagus, *Mt* middle thoracic esophagus, *Lt* lower thoracic esophagus

### Surgical procedure

Two- or three-field lymph-node dissection was performed in all patients. Thoracic operation was approached by robot-assisted, video-assisted thoracoscopic, or open esophagectomy, and abdominal operation was approached by laparoscopic, hand-assisted laparoscopic, or open surgery. The stomach or ileocolon was selected as the reconstructive organ. In cases of gastric tube reconstruction, a sub-total gastric tube with the greater omentum was created, and the tube was elevated via the retrosternal route. In ileocolonic reconstruction, the right colon and terminal ileum were mobilized. The ileocolic vessels and the marginal vessel on the ileal side were clamped. If arterial pulsation via the right colic artery was confirmed on the distal side of the ileocolic artery and there was no venous congestion, the ileocolon was deemed suitable for use as a reconstructive organ. The ileocolic artery and vein were dissected, the mesentery was incised with maintenance of vascular continuity to make the graft as straight as possible, and the graft was elevated via the retrosternal route. The stomach was the first choice as a reconstructive organ due to its simplicity, ease of elevation, and reliability [10]. However, in cases where the stomach cannot be used, such as in patients with a history of gastrectomy or coexisting gastric cancer, the ileocolon was selected. Additionally, the ileocolic reconstruction has the advantage of preventing reflux and preserving the gastric reserve function. Therefore, this graft was chosen for patients who were not among the cases listed below but requested this reconstruction: multiple colonic diverticula, multiple colonic polyps, previous colonic surgery, or an inadequate colonic vascular pattern. After dissection of the cervical esophagus, cervical anastomosis was performed by hand-sewn anastomosis.

### Postoperative management

If any postoperative events such as elevated inflammatory response or fever occurred, CECT was performed. Low-molecular-weight heparin was not routinely administered to prevent postoperative thrombosis.

### Diagnosis of UEDVT

**Fig. 2:**
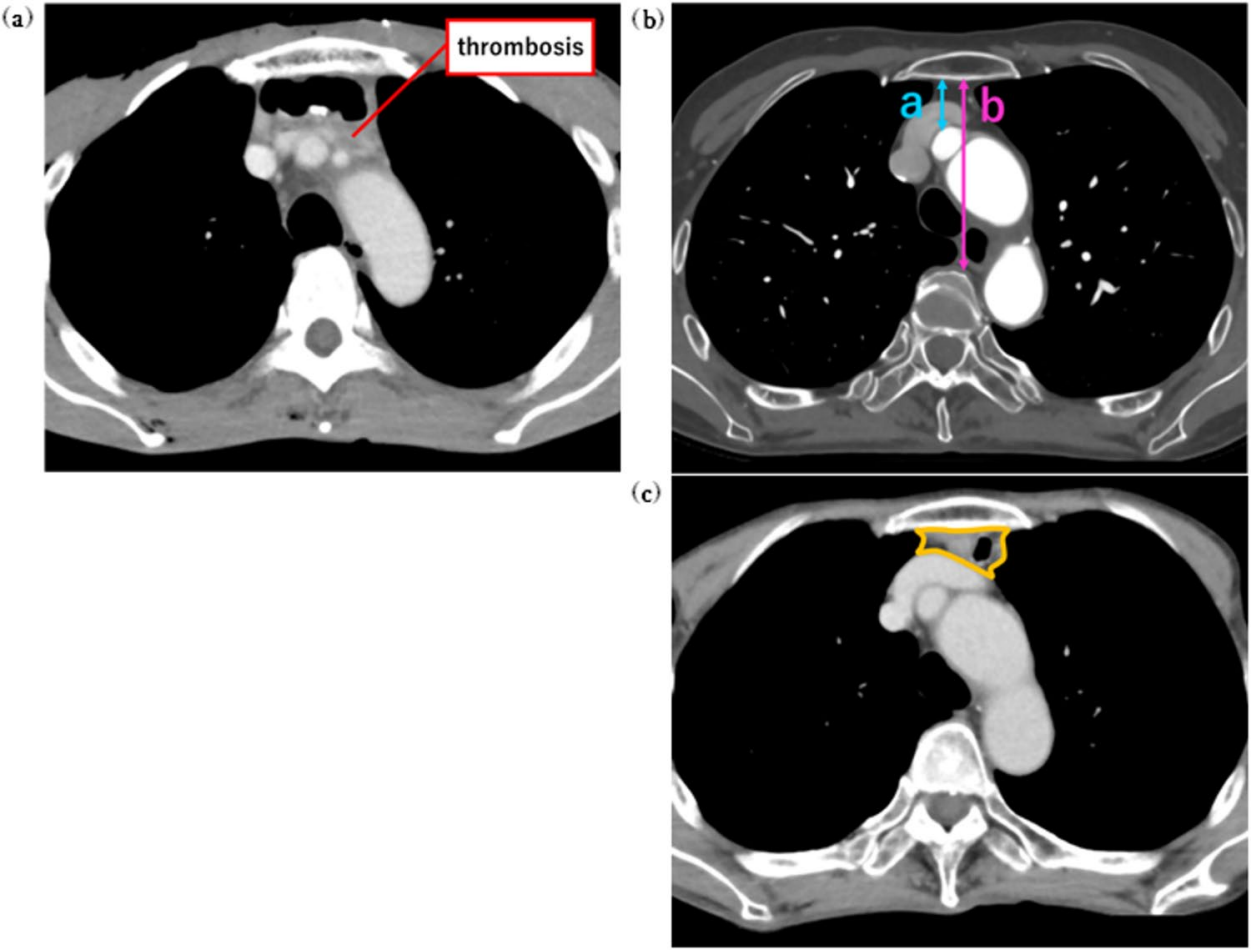
**a** Upper extremity deep vein thrombosis; **b** Measurement of the width of the retrosternal space. The distances from (**a**) the dorsal side of the sternum to the ventral side of the brachiocephalic artery and (**b**) the dorsal side of the sternum to the ventral side of the vertebra were measured at the level of middle of the left brachiocephalic vein were measured; **c** Cross-sectional area of the intestinal graft. To determine the volume of the intestinal graft, the total cross-sectional area of the graft and mesenteric fat at the level of middle of the left brachiocephalic vein was measured

UEDVT was defined as an intraluminal filling defect with poor contrast in the brachiocephalic, internal jugular, subclavian, axillary, external jugular, and brachial veins. All CT images taken within 2 years after surgery were independently interpreted by four doctors (Fig. 2a).

### Measurement of the width of the retrosternal space and cross-sectional area of the intestinal graft

In the preoperative CECT scan, (a) the distance from the dorsal side of the sternum to the ventral side of the brachiocephalic artery and (b) the distance from the dorsal side of the sternum to the ventral side of the vertebra were measured at the level of middle of the left brachiocephalic vein. The retrosternal ratio of (a) to (b) was calculated, and it was defined as the width of the retrosternal space (Fig. 2b).

To determine the volume of the intestinal graft, CT images taken within approximately one year after discharge from hospital were used. We measured the total cross-sectional area of the graft and mesenteric fat at the level of middle of the left brachiocephalic vein. This CT image was taken in a fasting state with the intestinal lumen sufficiently collapsed (Fig. 2c).

All of these measurements were taken at the same section based on the midpoint of the left innominate vein.

### Statistical analysis

The Mann–Whitney U test was used to compare non-normally distributed samples and Pearson’s Chi-square test was performed for intergroup comparisons. A *p* value of < 0.05 was considered statistically significant. Continuous variables were presented as the mean ± standard deviation (SD). All statistical analyses were performed using IBM SPSS Statistics ver. 25 (IBM Corp). The optimal cut-off point of the width of the retrosternal space for the prediction of UEDVT was determined, so that the Youden index (sensitivity + specificity − 1) would be maximized using receiver-operating characteristic (ROC) curve analysis.

## Results

### Postoperative UEDVT

Of the 75 cases, UEDVT was observed in 11 cases (14.7%). These included 8 males and 3 females with a median age of 64 years. The tumor location was the upper thoracic esophagus (Ut) in 3 cases, middle thoracic esophagus (Mt) in 2 cases, and lower thoracic esophagus (Lt) in 6 cases. The clinical stage (UICC 8th edition) was stage I in 3cases, stage II in 2 cases, stage III in 3 cases, and stage IV in 3 cases. Preoperative chemotherapy was performed in 7 cases. None of the patients had developed UEDVT before esophagectomy (Table 1). There were 4 cases in which UEDVT was observed within 3 weeks after surgery, all of which were discovered by CT scans for fever or high D-dimer levels. One of these 4 patients developed PE, and required prompt antithrombotic intervention. In the remaining 7 patients, thrombus was detected on the follow-up CT image several months after surgery (Table 2).

**Table 2:** Summary of the 11 patients who developed UEDVT after esophagectomy for esophageal cancer

No	Age	Gender	Location of UEDVT	PE	Symptom	Time of onset	Treatment
1	75	M	Left internal jugular vein	−	Fever	POD 11	Edoxaban
2	55	F	Left brachiocephalic vein	−	Fever	POD 15	Edoxaban
3	66	M	Left brachiocephalic vein	+	High D-dimer plasma	POD 7	Heparin → Edoxaban
4	63	F	Left internal jugular vein and subclavian vein	−	Fever	POD 21	None
5	68	M	Left brachiocephalic vein	−	None	7 months after surgery	Apixaban
6	52	M	Left brachiocephalic vein	−	Pitting edema appeared in the left upper limb	2 months after surgery	Warfarin
7	55	M	Left brachiocephalic vein	−	None	5 months after surgery	None
8	72	M	Left brachiocephalic vein	−	None	1 year after surgery	None
9	72	M	Left subclavian vein and brachiocephalic vein	−	None	5 months after surgery	None
10	64	M	Left brachiocephalic vein	−	None	4 months after surgery	None
11	63	F	Left subclavian vein	−	None	6 months after surgery	None

*PE* pulmonary embolism, *POD* post-operative day

Gastric tube reconstruction was performed in 54 cases and ileocolic reconstruction in 21 cases. UEDVT occurred only with gastric tube reconstruction (*p* = 0.02) (Table 1). The total cross-sectional area of the graft and mesenteric fat showed a statistically significant difference between the stomach and ileocolon (*p* < 0.01) (Table 3). Three-field lymph-node dissection was performed in 44 cases and two-field in 31 cases. There was no significant difference between the extent of lymph-node dissection and UEDVT (*p* = 0.085) (Table 1).

**Table 3:** Association between intestinal graft and cross-sectional area. *P* value was calculated by the Mann–Whitney U test

	Cross-sectional area	*P* value
Intestinal graft		
Stomach (n = 54)	855.53 ± 259.0	** < 0.01**
Ileocolon (n = 21)	350.98 ± 221.04	

### Retrosternal ratio

**Fig. 3:**
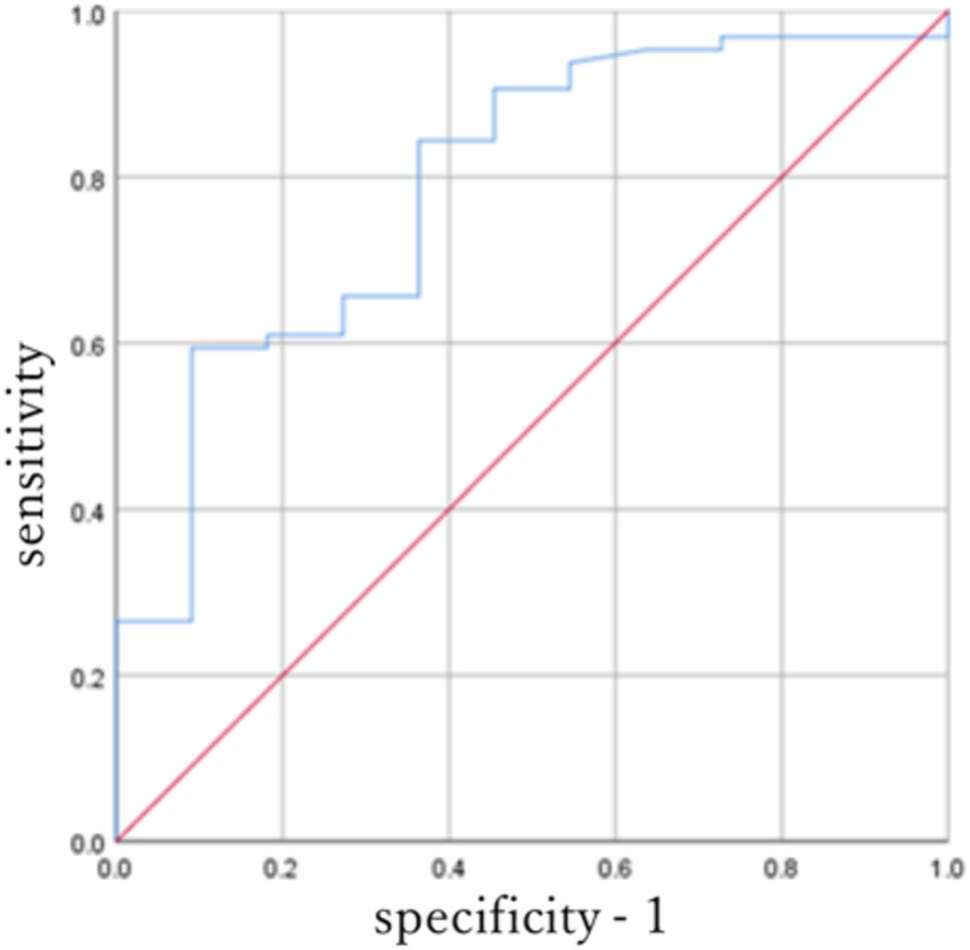
ROC curve analysis on the width of the retrosternal space. *ROC* receiver-operating characteristic

The retrosternal ratio was significantly different between the UEDVT and non-UEDVT groups (*p* = 0.002) (Table 1). The cut-off value for the width of the retrosternal space was estimated to be 0.16 [AUC: 0.789 (95% CI:0.65–0.93)], so that the Youden index (sensitivity + specificity–1) would be maximized. Using this cut-off value, UEDVT was detected with a sensitivity of 85.9% and a specificity of 63.6% (Fig. 3). Pearson’s chi-square test indicated a significant difference in the width of the retrosternal space between the UEDVT and non-UEDVT groups (*p* = 0.009) (Table 1).

### Central venous catheter

For patients who required chemotherapy, a PICC was inserted through the right or left ulnar brachial vein. There were no cases in which central venous catheter insertion was performed through the neck. The insertion side of the PICC was determined by the surgeon. There was a statistically significant difference between UEDVT and a history of PICC insertion from the left side (*p* = 0.025) (Table 4). In the UEDVT group, two patients without a history of PICC insertion from the left side did not undergo central venous catheter insertion during the perioperative period. There was no significant difference between an intestinal graft and a history of PICC insertion from the left side (*p* = 0.123) (Table 4).

**Table 4:** Comparison between the two groups on the history of PICC insertion from the left side

	History of PICC insertion from the left side		*P* value
	Yes	No	
Group			
UEDVT	9	2	**0.025**
non-UEDVT	28	36	
Intestinal graft			
stomach	30	24	0.123
ileocolon	7	14	

*PICC* peripheral central venous catheter

## Discussion

In this study, we attempted to identify the risk factors of UEDVT after esophagectomy. The analysis suggested that gastric tube reconstruction, a retrosternal ratio less than 0.16, and a history of PICC insertion from the left side may be risk factors for UEDVT. This is the first report to analyze the relationship between ileocolonic reconstruction and UEDVT and may serve as a baseline for future studies.

All patients who developed UEDVT had undergone retrosternal gastric tube reconstruction (*p* = 0.02). The total cross-sectional area of the graft and mesenteric fat was significantly larger for gastric tube reconstruction than for ileocolonic reconstruction (*p* < 0.01). CT scan after surgery showed that the left brachiocephalic vein was compressed by the gastric tube and its lumen was narrowed. Thus, it is thought that the thrombus was formed due to stasis of blood flow. On the other hand, UEDVT did not occur in ileocolonic graft reconstruction. We suspect that this is due to a smaller graft volume compared to the stomach, because only the ileum is located at the level of the left brachiocephalic vein in the ileocolonic reconstruction. No thrombus developed in the vein on the right side probably because the grafted organ did not compress these veins.

It has been reported that patients with a narrow retrosternal space are more likely to develop UEDVT [11, 12]. Using similar technique, we also calculated the retrosternal ratio, and it was defined as the width of the retrosternal space. As a result, we conclude that a retrosternal ratio less than 0.16 is a risk factor for UEDVT. In this study, no statistically significant differences were observed between the retrosternal space ratio and PICC insertion side. On the other hand, regarding the reconstruction method, there were significantly more cases with a wider retrosternal width in the ileocolic reconstruction group (*p* = 0.024). However, even when the analysis was limited to the gastric elevation reconstruction group, a statistically significant difference was observed in the retrosternal ratio and thrombus formation (*p* = 0.026). Therefore, we concluded that retrosternal gastric tube reconstruction should be avoided in cases with a retrosternal ratio of less than 0.16. In these previous two reports, CECT was performed in all cases within 1 week after surgery, and the incidence of UEDVT after esophagectomy was reported to be 15.9% to 16.7%. As we did not routinely perform CT scan in the early postoperative period, it is possible that there might have been some cases in which the occurrence of UEDVT was overlooked.

Although no statistically significant difference was observed for lymph-node dissection (*p* = 0.085), UEDVT more frequently occurred in three-field lymph-node dissection. Three-field dissection has a higher incidence of PE compared to other surgical procedures for esophageal cancer, and cervical lymph-node dissection is an independent risk factor that significantly increases the incidence of VTE in the surgical treatment of esophageal cancer [13, 14]. In the lymph-node dissection of the neck, intraoperative manipulation of the internal jugular vein or subclavian vein may induce vascular damage and promote thrombus formation.

Four patients developed UEDVT within 3 weeks after surgery. The onset was fever in three patients and high D-dimer plasma in one patient (Table 2). Since fever is an important factor that induces thrombus, it is possible that the cause of UEDVT was not the reconstruction method or the narrowness of the retrosternal space but a change in vital signs [15]. D-dimer level is also an important marker that reflects the dynamics of postoperative coagulation factors [16]. However, since we did not routinely measure D-dimer levels, we were unable to perform an analysis of D-dimer levels. In recent years, prophylactic anticoagulant therapy has been widely performed to prevent postoperative VTE, but we do not administer it because of the postoperative bleeding [17]. If low-molecular-weight-heparin is administered during the perioperative period, the occurrence of UEDVT may be suppressed.

The occurrence of UEDVT was also observed in the late postoperative period. In Case No. 5, a metastatic lung tumor was detected 7 months after surgery, and two courses of pembrolizumab plus FP chemotherapy were performed (Table 2). In one of these courses, the PICC was inserted through the left ulnar cutaneous vein, and UEDVT was discovered incidentally on CT scan after chemotherapy. In Case No.6, local recurrence was noted 2 months after surgery, and 6 courses of FP regimen and 11 courses of nivolumab therapy were administered (Table 2). In five of these cases, the PICC was inserted from the left side. After chemotherapy, marked pitting edema appeared in the left upper limb, and the patient was diagnosed with UEDVT. As mentioned above, after gastric tube reconstruction, the brachiocephalic vein is already compressed and DVT is likely to occur. In this study, the incidence of UEDVT was significantly higher in patients with a history of PICC insertion from the left side (*p* = 0.025). Furthermore, a central venous catheter is a risk factor for VTE, and treatment with cisplatin-based chemotherapy have been reported to increase the incidence of VTE [18]. Even though the cancer-bearing state is a risk factor for thrombosis and the presence of a recurrent lesion itself may have caused UEDVT, but it may be necessary to avoid retrosternal gastric tube reconstruction for cases with a high possibility of recurrence after surgery and needing postoperative chemotherapy. Also, if chemotherapy is to be administered postoperatively, PICC insertion should be approached from the right side. Furthermore, retrosternal gastric tube reconstruction should be avoided in patients with a history of left-sided PICC insertion prior to esophageal cancer resection.

Limitations of our study include its retrospective nature and the small number of patients in a single institution. The timing of CT scans could not be synchronized. As a result, there were variations in the rate of change in the nutritional status and body composition of postoperative patients, which might have led to a lack of statistical accuracy in the values for the cross-sectional area of the intestinal graft. There were several cases in which the presence of UEDVT was not recognized and therapeutic intervention was not performed. In addition, because UEDVT did not occur in the ileocolic reconstruction group, accurate multivariate analysis was not possible due to statistical error. However, binary logistic analysis showed that a retrosternal ratio of less than 0.16, PICC insertion from the left, and graft cross-sectional area were risk factors for UEDVT (*p* = 0.004, odds ratio (OR) 20.843; 95% confidence interval (95% CI) 2.607–166.655, *p* = 0.006, OR 28.067; 95% CI 2.546–309.358, *p* = 0.035, OR 1.004; 95% CI 1.000–1.008, respectively). When a similar analysis was performed on the gastric elevation reconstruction group only, a retrosternal ratio of less than 0.16 and PICC insertion from the left were independent risk factors (*p* = 0.002, OR 22.068; 95% CI 3.089–157.638, *p* = 0.023, OR 0.116; 95% CI 0.018–0.740, respectively). Therefore, we suggest that gastric tube reconstruction, a retrosternal ratio less than 0.16, and a history of PICC insertion from the left side are risk factors for UEDVT. This study is the first report comparing retrosternal gastric tube reconstruction with ileocolonic reconstruction for UEDVT after esophageal cancer surgery. It is necessary to accumulate more cases and select a reconstruction that is more suitable for each patient's background.

## Data Availability

The data that support the findings of this study are available from the corresponding author upon reasonable request. However, the data are not publicly available due to patient privacy concerns or ethical restrictions.
